# Natural Recovery and Planned Intervention in Coastal Wetlands: Venice Lagoon (Northern Adriatic Sea, Italy) as a Case Study

**DOI:** 10.1155/2014/968618

**Published:** 2014-07-13

**Authors:** Chiara Facca, Sonia Ceoldo, Nicola Pellegrino, Adriano Sfriso

**Affiliations:** Department of Environmental Sciences, Informatics & Statistics, Ca' Foscari University of Venice, Calle Larga Santa Marta 2137, 30123 Venice, Italy

## Abstract

The goals of conservation and sustainable use of environmental ecosystems have increased the need for detailed knowledge of ecological evolution and responses to both anthropogenic pressures and recovery measures. The present study shows the effects of natural processes and planned intervention in terms of reducing nutrient inputs in a highly exploited coastal lagoon, describing its evolution over a 16-year period from the late 1980s (when eutrophication was at its peak) until 2003. Changes in nutrient and carbon concentrations in the top layer of sediments were investigated in parallel with macroalgal and seagrass biomass in the most anthropized basin of Venice Lagoon in four surveys conducted in accordance with the same protocols in 1987, 1993, 1998, and 2003. A pronounced reduction in trophic state (mainly total nitrogen, organic phosphorus, and organic carbon concentrations) and macroalgal biomass was recorded, together with the progressive expansion of seagrass meadows. General considerations are also made on the effects of Manila clam farming and the shift from illegal to managed clam farming.

## 1. Introduction

Since the mid-20th century, in addition to land reclamation, coastal areas have been seriously affected by eutrophication, compromising environmental equilibria and species diversity. The accentuation of these problems has been studied and described from many points of view, considering human activities, hydrodynamic and morphological features of basins, and so forth [1–3]. Partly because ecosystems have been shown to vary widely in terms of their sensitivity to nutrient enrichment [2], it may be difficult to distinguish between natural nutrient concentrations and anthropogenic inputs. However, the loss of seagrass beds appears to be a common pattern in coastal areas affected by eutrophication [4–6]. Although little historical information is available on seagrass distribution, a general decline in coverage has been reported for most European coastal waters and less than 15% of shorelines can be considered to be in good condition [7]. On a global scale, seagrass loss due to anthropic activities is estimated at 60% [5].

Since 1971, when the Ramsar convention [8] was signed, a number of protection policies have been adopted at the European level for reducing the effects of anthropogenic disturbances and for the conservation and recovery of marine and coastal natural heritage (see Table  1 in [7]). The latest EU regulations are set out in the Water Framework Directive (WFD 2000/60/EC) and the Marine Strategy Directive (2008/56/EC), which establish common objectives for member states in the assessment and recovery of aquatic ecosystems. In 1998, a Spanish National Ramsar Report estimated that 56.6% of coastal wetlands had been lost, and restoration processes were planned by regional authorities in deeply degraded ecosystems, such as the brackish lagoon of Senillar de Moraira (Mediterranean Valencia Region, Spain [9]). In this case the intervention plan mainly consisted of rebuilding the hydrogeomorphological features but the remaining anthropogenic pressures (dense urban and recreational areas) strongly compromised the restoration processes and the ecosystem functioning did not appear self-sustainable, requiring continuous maintenance measures [9]. The need to combine restoration with the removal of the causes of degradation in order to favour the self-maintenance of natural processes is a major challenge [9]. Similar but more encouraging results were observed in Veerse Meer coastal lagoon (Netherlands, North Sea), where the measures consisted of opening up a connection between the enclosed brackish lagoon and the adjacent marine bay [10]. Rapid and constant improvement of water quality was seen, with reductions in nutrient concentrations and frequency of algal blooms and an increase in water transparency. Nevertheless, corresponding positive effects were not observed for the composition and abundance of macrofauna, which may have been affected by changes in hydrodynamism and consequent sediment resuspension [10]. As with Senillar de Moraira, in this latter case the restoration consisted of modifying the hydrological features with no direct measures to reduce nutrient inputs, which remain a major threat to aquatic ecosystem functioning. Better results were observed in Tampa Bay (Florida, USA), where cyanobacteria blooms became less frequent and seagrasses recolonized the bay following a 10-fold reduction in annual wastewater N loading [4].

Assessing recovery measures may be complicated by the need for a good understanding of interactions between stressors and by the different sensitivity of coastal ecosystems to anthropogenic pressures. However, most of the conceptual models describing eutrophication processes associate the presence of well-developed seagrass beds with pristine conditions [5, 6, 11] and they are recognized as indicators of good/high ecological quality (*sensu* Water Framework Directive [12]). Hence, the presence/absence of angiosperms can represent a starting point for verifying the efficiency of coastal management and gaining a better understanding of the complexity of transitional ecosystems.

**Figure 1:**
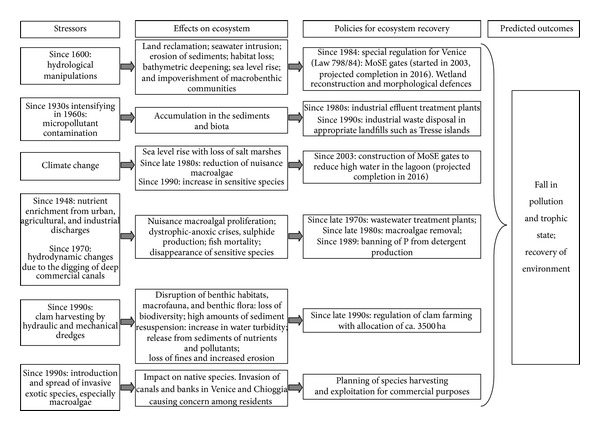
Scheme of main stressors (categories in accordance with [4]) affecting Venice Lagoon, effects on ecosystem, policies adopted, and predicted outcomes.

On the basis of the conceptual model described in [4], we identified the stressors affecting Venice Lagoon (Northern Adriatic Sea, Italy) and the main effects on the ecosystem with reference to the available literature [13].  Figure 1 highlights the multiplicity of human pressures affecting this particular basin. For each stressor, the environmental consequences and resulting policies are cited. The management of Venice Lagoon is of international concern due to the historical heritage of the city, increasingly threatened by flood damage. Considerable sums have thus been invested in the MoSE gates (experimental electromechanical module), which will close off the inlets connecting the lagoon to the sea at times of “Acqua Alta” (high tide) thereby reducing the environmental impact and erosion (http://www.salve.it/uk/soluzioni/acque/f_avanzamento.htm; last access November 15, 2013).

**Figure 2:**
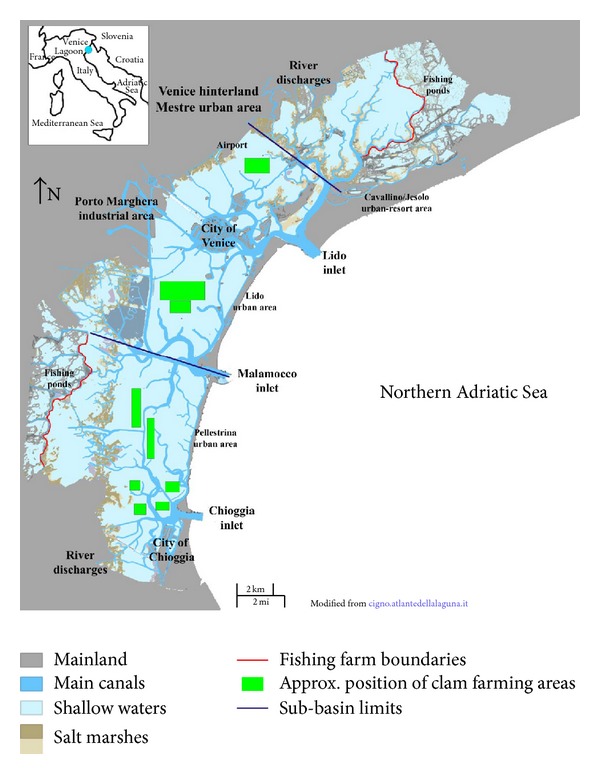
Study area: Venice Lagoon. Main anthropogenic pressures indicated in bold black. Solid blue lines separate lagoon into three morphological basins. Details of clam farming areas in [27].

In the early 1970s industrial inputs of nitrogen and phosphorus to the lagoon were estimated to be 8200–10060 and 1100–1900 tonnes y^−1^, respectively [14]. These nutrient loads favoured the proliferation of nuisance macroalgae, which in the central lagoon basin produced one of the highest standing crops in the world (between 5 and 20 kg fwt m^−2^ over a surface area of 66 km² [15]). Our first survey in the central part of the lagoon in 1987 was carried out under such conditions, when seagrass beds had already disappeared. By the end of the 1990s, industrial inputs of nutrients had decreased by about one order of magnitude [16], thanks to the positive effects of waste water treatment plants and progressively falling industrial production. However, the disappearance of nuisance macroalgal blooms in the early 1990s depended on a combination of several factors, including unfavorable weather conditions, increasing water turbidity, and grazing pressure [15, 17, 18]. The complete absence for many years of dystrophic-anoxic crises and the strong reduction of macroalgae on the bottom favoured the spread of the Manila clam* Tapes philippinarum* Adams & Reeve (introduced into the lagoon in 1983 for aquaculture purposes), reaching biomass of up to 7.45 kg m^−2^ [19]. In a few years the high clam biomass became an important economic resource, with production peaking at ca. 40,000 tonnes in 1998 (the year of our third survey), mainly harvested by unauthorized fishermen [20]. The continuous resuspension of sediments caused by harvesting techniques strongly affected the benthic habitat, reducing light transmission and altering sediment compactness and texture [21, 22] as well as oxygenation [23]. A further result of sediment resuspension was that nutrient [24] and pollutant [25, 26] concentrations decreased. Due to the dramatic impact on the lagoon ecosystem and the risks for human health (clams were illegally harvested in polluted areas), clam farming was regulated by restricting it to specific areas (Figure 2). About 3500 ha were assigned by the local administration [20], reducing the impact of this activity on surface sediments and favouring their recolonization by seagrasses.

This paper aims to describe

- trophic conditions throughout the lagoon before the start of work on the MoSE project in 2003, thereby providing a benchmark against which future changes in ecological features can be assessed,
- ecological evolution over a 16-year period, moving from the peak eutrophication of the late 1980s, through the period of uncontrolled clam harvesting activities, until the attempts at sustainable clam farming.

## 2. Materials and Methods

### 2.1. Study Area Characteristics

Venice Lagoon (Figure 2) is located in the Northwestern Adriatic Sea. It has a surface area of ca. 550 km^2^ of which ca. 432 are subject to tidal exchange. During each tidal cycle (12 hr) ca. 60% of the lagoon's waters are exchanged with the sea through three large inlets (400–900 m wide and 8–20 m deep). Except for the main channels, the mean water depth is approximately 1 m. The mean tidal amplitude is ca. 60 cm [28], but during syzygy tides it ranges between 1 and 1.5 m. The lagoon receives industrial and treated urban wastewaters from a large drainage network (1839 km^2^ [14]), as well as untreated urban sewage from the historical city of Venice, the islands of Lido and Pellestrina, and the city of Chioggia and its hinterland (ca. 300,000 inhabitants).

With reference to morphological features the lagoon is divided into three basins (Figure 2):

- the northern basin, delimited to the south by the Burano and Torcello salt marshes, which has few inhabited areas, little naval traffic, and low hydrodynamism;
- the central basin, bounded to the north by the Burano and Torcello salt marshes and to the South by the Malamocco-Marghera Ship Canal. This basin is characterised by the highest anthropogenic pressures due to urban, industrial, and maritime activities;
- the southern basin to the south of the Malamocco-Marghera Ship Canal, where human pressures are relatively low and mainly related to clam farming. This is the largest of the three basins.

### 2.2. Sampling and Analytical Methods

**Figure 3:**
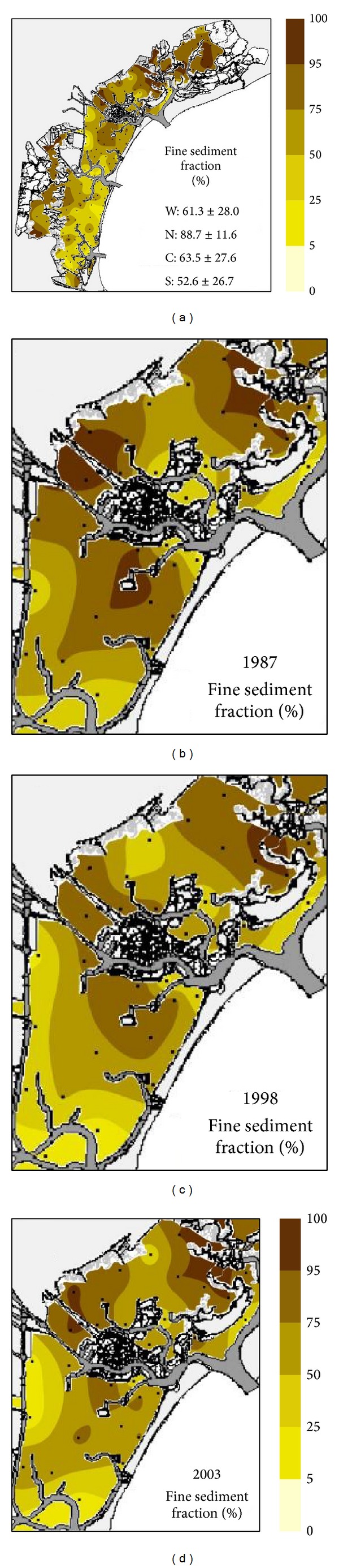
Spatial distribution of fine sediments (fraction < 63 *μ*m) in 2003 (a). Mean values and standard deviations shown on map for whole lagoon (W) and northern (N), central (C) and southern (S) basins. Other maps indicate distributions in 1987 (b), 1998 (c), and 2003 (d) in central basin.

**Figure 4:**
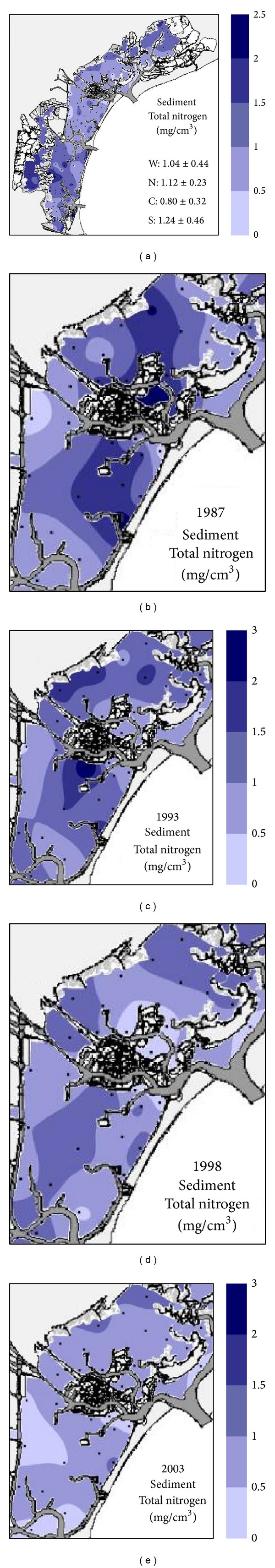
Spatial distribution of TN in 2003 (a). Mean values and standard deviations shown on maps for whole lagoon (W) and northern (N), central (C) and southern (S) basins. Other maps indicate distributions in 1987 (b), 1993 (c), 1998 (d), and 2003 (e) in central basin.

**Figure 5:**
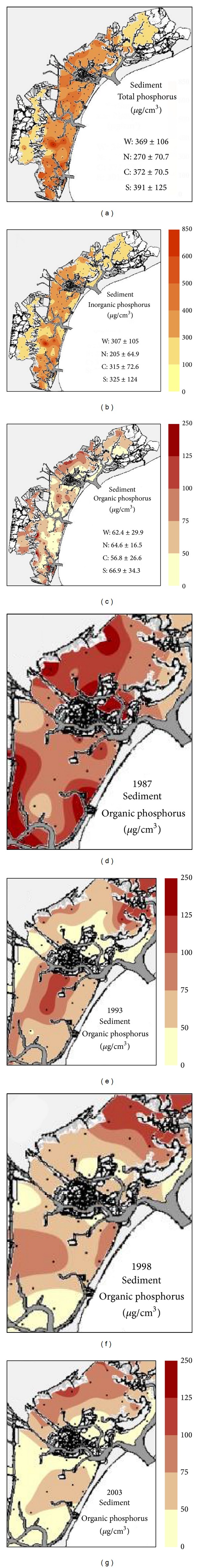
Spatial distribution of phosphorus concentrations in 2003: total phosphorus (a), inorganic phosphorus (b), and organic phosphorus (c). Mean values and standard deviations shown on maps for whole lagoon (W) and northern (N), central (C) and southern (S) basins. Other maps indicate distributions in 1987 (d), 1993 (e), 1998 (f), and 2003 (g) in central basin.

**Figure 6:**
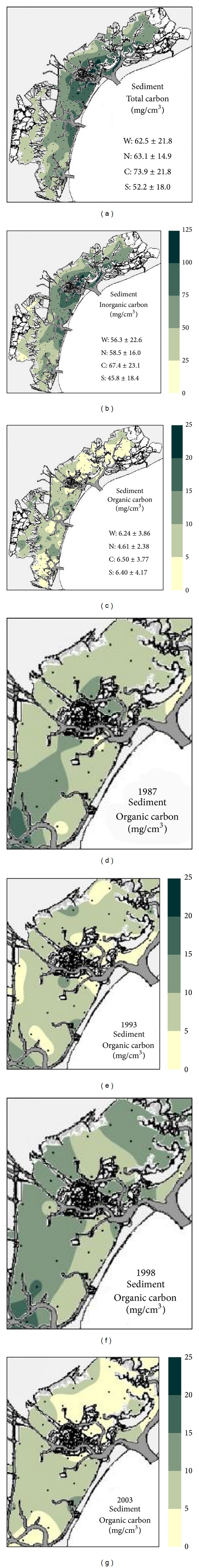
Spatial distribution of carbon concentrations in 2003. Total carbon (a), inorganic carbon (b), and organic carbon (c). Mean values and standard deviations shown on maps for whole lagoon (W) and northern (N), central (C) and southern (S) basins. Other maps indicate distributions in 1987 (d), 1993 (e), 1998 (f), and 2003 (g) in central basin.

In the last week of June and the first few days of July 2003, sampling was conducted throughout the lagoon. A total of 165 sites were sampled, including the 31 of previous surveys (June 1987, June 1993, and June 1998). The site locations are displayed in Figures 3, 4, 5, and 6. They are located exclusively in areas of the lagoon that are subject to tidal exchange; enclosed fish-farming ponds were not considered as they are managed as private properties and regulated in accordance with the needs of fish-farming. Indeed, they are not included in ecological restoration policies because it is recognized that the benefits of such uses need to be retained (*sensu* WFD).

In order to obtain comparable results, the sampling procedures and analytical protocols were the same as those of the previous 3 surveys (1987, 1993, and 1998).

Sediment samples of the top 5 cm layer were obtained by mixing 3-4 cores collected by a Plexiglas corer (i.d. 10 cm). The sites selected for our investigations are representative of the study areas and are characterized by uniform sediment texture. They are distant from channel edges or bottom discontinuities, and therefore intrasite variability is low and the mixing of 3-4 cores guarantees variability of less than 5%.

The water parameters near the bottom were

- temperature (*T*) and pH, measured by portable pH-meter (model HD 8705, Delta OHM, Padua, Italy) equipped with a thermocouple (precision 0.1°C);
- chlorinity, determined argentometrically in the laboratory using a modified version of the Knudsen method [29]. Salinity (*S*) was calculated in accordance with the formula Cl^−^ × 1.805 + 0.03;
- dissolved oxygen, measured by portable oximeter (Oxi 196, Wissenschaftlich-Technische Werkstätten GmbH, Weilheim, Germany). The data are reported as the oxygen saturation percentage (OS), taking account of water temperature and salinity;
- light extinction depth, measured by Secchi disk (SD).

In the top 5 cm sediment layer the parameters were

- mud content: the fine sediment fraction, that is, <63 *μ*m (fines), was determined by wet sieving [30] in order to classify the lagoon sediments in accordance with [31] into 6 textural classes: <5% sand; 5–25% slightly muddy sand; 25–50% muddy sand; 50–75% sandy mud; 75–95% slightly sandy mud; and >95% mud;
- the amount of dry sediment by unit of volume (dry density: g dwt cm^−3^). Sediment dry density (SDD) was used to normalise nutrient concentration by volume;
- inorganic phosphorus (IP), measured by sediment sonication in 1 N HCl of finely pulverized freeze-dried samples (ca. 0.4 g) in accordance with Aspila et al. [32]. The resulting solutions were analyzed spectrophotometrically in accordance with Strickland and Parsons [33]. TP was obtained using the same procedure after 2 hrs combustion at 550°C and the OP fraction was calculated from the difference. All measurements were replicated until the experimental error was <5%;
- TN, TC, and IC concentrations, measured by Carlo Erba CNS Autoanalyser, mod. NA 1500. TN and TC were analyzed directly after sediment powdering and IC was determined by combusting samples for 2 hrs at 440°C in order to eliminate most of the organic matter with negligible loss of carbonates [34]. For mainly carbonatic sediments (50–80%) as in the current case, this temperature has been found to yield the closest results to those obtained with the acidification method described by Froelich [35]. Both methods are susceptible to systematic errors arising from the elimination of OC and IC, but the combustion method is preferable because the acidified samples may destroy some of the components of the CNS-analyzer. OC was determined from the difference. All analyses were replicated on different days till the experimental error was <5%.

The concentrations of nutrients in the top layer of sediments were normalised with reference to dry sediment density in order to obtain actual nutrient loads per unit of volume (cm³). The raw concentrations expressed per unit of weight (g) reflect the substance inputs, whereas the normalized values highlight how they are distributed in the environment in relation to sediment characteristics [24].

The results were mapped using the Surfer system (Golden Software Inc., 1993–2000), applying the kriging method.

In accordance with the previous sampling protocols [18] and literature data on the annual growth of each species [36–38], macroalgae were recorded within 6 biomass ranges: 0.01–0.1, 0.1–0.5, 0.5–1.0, 1.0–5.0, and 5.0–10.0 kg fwt m^−2^, although in the southern lagoon the highest range was only 1.0-2.0 kg fwt m^−2^. The three seagrass species (*Cymodocea nodosa *(Ucria) Asherson,* Zostera marina* Linnaeus, and* Nanozostera noltii* (Hornemann) Tomlinson* et* Posluzny) were sampled separately, with biomass (shoots + roots and rhizomes) recorded within four coverage ranges: 0–25%, 25–50%, 50–75%, and 75–100%, corresponding to the following biomass intervals: 0–1.9, 1.9–3.8, 3.8–5.6, and 5.6–7.5 kg fwt m^−2^ for* C. nodosa*, 0–1.5, 1.5–3.0, 3.0–4.5, and 4.5–6.3 kg fwt m^−2^ for* Z. marina,* and 0–1.4, 1.4–2.7, 2.7–4.1, and 4.1–5.4 kg fwt m^−2^ for* N. noltii*.

### 2.3. Statistical Analyses

Before any statistical analysis, the distribution of each variable was tested for normality by the Kolmogorov-Smirnov test (*p* < 0.05), variables with non-parametric distribution being pre-treated by log-transformation. All variables were then standardized (mean 0 and variance 1) in order to reduce the variability arising from the different units of measurement.

Non-parametric Spearman's correlation coefficients (*r*_*s*_) were considered significant at a *p* value of <0.001, data being processed using STATISTICA 7.1 (StatSoft srl). Redundancy analysis (RDA) was carried out on a matrix of 165 cases (sites) and 11 independent variables (salinity, pH, dissolved oxygen, mud content, inorganic and organic carbon, inorganic and organic phosphorus, total nitrogen, and macroalgal and seagrass biomass) to determine the part of the variation in species composition explained by environmental variables, data being processed using CANOCO 5.

To verify changes occurring between the surveys carried out in 1987, 1993, 1998, and 2003 in the central lagoon, 31 sites were compared by two-way ANOVA (*p* value <0.05). The data were considered to be comparable, as the sampling position and protocols, as well as the laboratory analytical procedures, were exactly the same.

## 3. Results

### 3.1. Findings for the Whole Lagoon (2003)

#### 3.1.1. Physico-Chemical Parameters

The descriptive statistics for the main physico-chemical parameters are reported in Table 1. The central basin showed lower salinity and pH than the others. It also had the worst underwater light transmission, on the landward side.

The lagoon is characterised by sandy mud (Table 1; Figure 3(a)) except for the northern basin, where slightly sandy mud prevails (Figure 3(a)). It is important to note that areas of sand or slightly muddy sand were not only located on the seaward side of the lagoon, where they naturally occur in transitional systems, but also close to the industrial area, into which a river once flowed, being diverted in the 15th century (Figure 3(a)).

#### 3.1.2. Nutrient Concentrations

The concentration distributions of total nitrogen (TN), total, inorganic, and organic phosphorus (TP, IP, OP), and total, inorganic, and organic carbon (TC, IC, OC) are shown in Figures 4, 5, and 6, together with means and standard deviations for each parameter.

The northern and southern basins displayed a clear TN gradient, with values descending from landward to seaward, whereas a more homogeneous distribution was observed in the central one (Figure 4(a)). TP distribution differed from that of TN, the highest concentrations being recorded in the central basin (Figure 5(a)). Although OP had patchy distribution, a decreasing seaward gradient was observed almost throughout the lagoon (Figure 5(c)). OP accounted for between 0.9 and 59.5% of TP and was 18% on average.

TC and IC concentrations were highest in the central basin around the city of Venice, decreasing towards the other basins and towards the landward side (Figures 6(a) and 6(b)). The sites closest to the three seaward inlets, above all in the Lido inlet, showed high IC concentrations due to the dominance of dolomite (and to a lesser extent calcite) of fluvial origin [39]. In contrast, the lowest concentrations (<25 mg cm^−3^, Figure 6(b)) were recorded in the confined sites of the southern basin. OC was <5 mg cm^−3^ in most of the lagoon, exceeding 5 mg cm^−3^ only in the sites north of Venice, close to Chioggia and in the salt marshes of the southern basin (Figure 6(c)).

#### 3.1.3. Macrophyte Distribution

**Figure 7:**
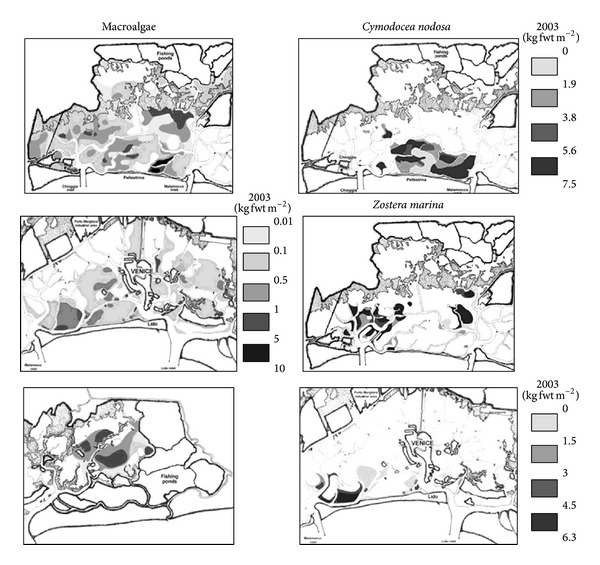
Macrophyte biomass distribution in 2003. Modified from [18].

Macroalgal biomass was more abundant in the southern basin (Table 1), where it was found at most of the sites (Figure 7). In the northern and central part of the lagoon, some sites, mainly on the landward side, had no macroalgal coverage at all.* C. nodosa* and* Z. marina* were recorded in the southern basin and in a few sites in the central one (Figure 7), showing a progressive expansion of meadows [18]. In the northern basin seagrass coverage was negligible.

#### 3.1.4. Statistical Analyses

The Kolmogorov-Smirnov tests highlighted non-normal distribution for salinity, pH, oxygen saturation, and macrophyte biomass, for which values were log-transformed.

Correlations of *r*_*s*_ ≥ |0.26| were highly significant (*p* < 0.001). Salinity was not found to be related to nutrient and carbon distribution but was positively correlated with temperature, pH, and macrophyte biomass. Among benthic producers, seagrasses (but not macroalgae) were negatively correlated with fines and OP. Fines were also the main driver for nutrient distribution (being negatively correlated with TP and IP and positively correlated with OP and TN). TP and TC variations both depended almost entirely on changes in the inorganic fractions, Spearman's coefficients being 0.95 and 0.98, respectively. Lastly, the distributions of OP and OC were positively correlated with TN.

**Figure 8:**
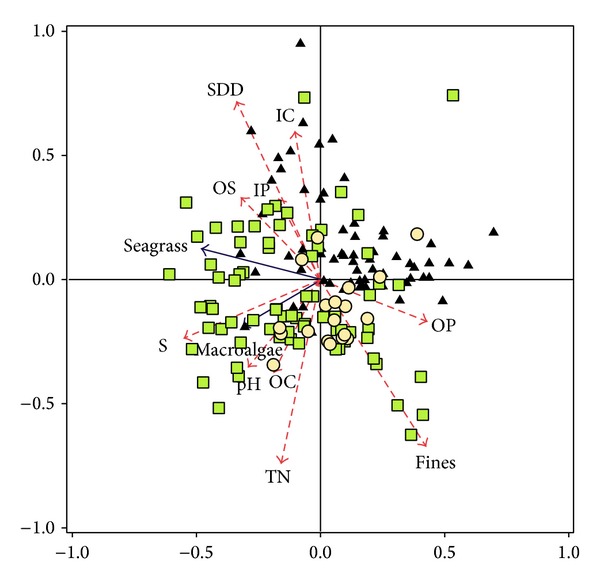
Redundancy analysis (RDA) for dataset including 165 observations in whole lagoon, 2 biotic factors (macroalgae and seagrass), and 9 environmental variables: *S* = salinity, pH, OS = oxygen saturation, fines = sediment fine fraction, TN = total nitrogen, IP = inorganic phosphorus, OP = organic phosphorus, IC = inorganic carbon, and OC = organic carbon. Black triangles represent central basin samples; green squares represent southern basin samples; yellow circles represent northern basin samples. First and second axes explain 50.7% and 16.7% of variation, respectively.

Two axes explained 67.40% of cumulative variation in the RDA. Axis 1 (Figure 8) tends to isolate most of the southern basin sites from most of the central basin sites. According to the environmental data, axis 1 separates sites with higher OP content (on the right) from sites with more seagrasses and macroalgae and higher salinity, pH, OS, and IP (on the left). Axis 2 points to a partition based mainly on IC, SDD, TN, and fines (with Spearman rho values of 0.60, 0.72, −0.75, and −0.70, resp.). Northern basin sites are mostly grouped in the lower part of the graph, being characterized by muddy sediments.

### 3.2. Changes in the Central Basin from 1987 to 2003

Sediment samples collected in 31 sites located in the central lagoon in 2003 were compared with samples taken from the same sites in 1987, 1993, and 1998. Mean salinity, fines, TC, IC, and TP did not change significantly during the study period (ANOVA per time *p* > 0.05; Table 2), but the maps and the ANOVA per site (*p* < 0.001) showed important changes in spatial distribution, mainly that of fines (Figures 3(b), 3(c), and 3(d)), which decreased in landward areas. All other parameters displayed highly significant changes both on spatial and temporal scales (two-way ANOVA *p* < 0.001; Table 2). Specifically, there was a decreasing trend of macroalgal biomass and consequently a reduction in pH and oxygen saturation (Table 2). Concerning sediment concentrations, the most important variations were observed for TN, OP, and OC (Figures 4(b), 4(c), 4(d), 4(e), 5(b), 5(c), 5(d), 5(e), 6(b), 6(c), 6(d), and 6(e); Table 2). The mean TN value in 2003 was roughly half that of 1987 and both the maximum and minimum values were 2.5 times lower than 1987. On a spatial scale, the central basin displayed almost homogeneous TN distribution, with most values <1 mg cm^−3^ (Figures 4(b), 4(c), 4(d), and 4(e)). In previous years a more patchy distribution highlighted areas of urban discharge such as the city of Venice. TN reduction was progressive throughout the years (Table 2), whereas OP decreased mainly between 1987 and 1993, the period of macroalgal biomass decline (Table 2). On a spatial scale the most significant OP depletion was recorded south of Venice, with values uniformly below 50 *μ*g cm^−3^ in 2003 (Figures 5(b), 5(c), 5(d), and 5(e)). OC fluctuations did not follow a univocal trend (Table 2), being highest in 1998 and lowest in 2003. In the latter case, the city of Venice divided the central basin into two areas: the northern one, characterized by values of <5 mg cm^−3^, and the southern one with concentrations ranging between 5 and 10 mg cm^−3^ (Figures 6(b), 6(c), 6(d), and 6(e)).

## 4. Discussion

Pristine and unimpacted coastal ecosystems that could be used as benchmarks for assessing recovery of degraded areas have almost disappeared [7]. However, time series data [40] can make it possible to trace environmental evolution, showing how ecosystems change in relation to anthropogenic pressures and subsequent recovery measures. Although coastal lagoon environmental restoration seeks to reduce nutrient enrichment, it mainly entails morphological measures, with no action taken to reduce nutrient inputs [9, 10]. Case studies show that such a strategy is not sufficient to restore ecosystem self-sustainability and that effective limits on anthropogenic discharges are necessary. This is confirmed by the case of Tampa Bay (Florida, USA), where the reduction of nutrient inputs enabled seagrass recolonization in about a decade [4]. Borja et al. [41] listed 50 subtidal and intertidal coastal ecosystems affected by different anthropogenic pressures (wastewater discharge, eutrophication, fish-farming and trawling, sewage sludge disposal, etc.) and indicated the time span for recovery after restoration or removal of pressures. Data on the recovery times of macroalgae and seagrasses are available for only 7 cases, of which only 2 involve areas affected by eutrophication [41].

In the present paper the ecological evolution of Venice Lagoon over a 16-year period was related both to measures to reduce nutrient inputs and anthropogenic impact and to natural changes such as the fall in macroalgal biomass mainly as a result of climate change [42].

In the late 1980s biogeochemical cycles were mostly driven by nuisance macroalgal blooms, with huge nutrient uptake and massive biomass decay. Management policies (wastewater treatment plants, banning of phosphate compounds from detergent formulation by the Decree of the President of the Italian Republic n. 250/1989, mechanical collection of macroalgal biomass) seeking to limit eutrophication and its effects were successful only after nuisance macroalgal proliferation was compromised by unfavourable weather conditions [15, 18]. A significant fall in water column nutrient levels had already been seen by the 2000s [43, 44]. This paper highlights nutrient reductions in surface sediments, especially for TN, OP, and OC (Table 2; Figures 4(e), 5(e), and 6(e)). This strong decrease depended mainly on the elimination of huge biomass production and collapse cycles and on the start of intense clam harvesting in the mid-1990s. However, intense clam harvesting by heavy hydraulic and mechanical dredges also caused disruption of benthic habitats, persistent water turbidity, resuspension and redistribution of toxic contaminants (Figures 3(b) and 3(c)), and loss of fines (Table 2), further affecting primary production [45].

The loss of TN in the top layer of sediments in the period when clam density was high and the intense harvesting had not yet started may be also explained by sediment bioperturbation due to clam burrowing, which plays a significant role in the nitrogen cycle, favouring the release of ammonium and orthophosphates and the intensification of denitrification processes [46, 47]. In the central basin, the most severely affected by clam harvesting, industrial and urban discharges, and naval traffic, TN distribution was <1 mg cm^−3^ almost everywhere. In contrast, areas less affected by clam harvesting, such as the southern lagoon, maintained higher TN concentrations, confirming the hypothesis of significant loss driven by sediment perturbation. In fact, pore water released by sediment perturbation contains ammonium and orthophosphates at concentrations 2-3 orders of magnitude higher than the water column [48].

Likewise, the disappearance of high macroalgal biomass and the sediment washing arising from clam catching strongly reduced OP, whereas IP remained almost the same (Table 2). The depletion of phosphorus in the water column [43] has made it the main limiting factor for phytoplankton and macroalgal growth. Although phosphorus concentrations have also fallen in surface sediments, the overall effect has been to favour the spread of seagrasses, as observed in 2003 and subsequent years, since these organisms can still tap into sedimentary phosphorus via their root-rhizome system whereas macroalgal biomass decreases. Seagrasses, especially* C. nodosa* and* Z. marina*, are now the lagoon's main producers [18], suggesting rapid restoration of good/high ecological conditions. Even though the most confined areas of the lagoon have not yet been recolonized by seagrasses, because of the absence of seeds or rhizomes, these results show that the fall in nutrient concentrations has significantly favoured progressive environmental recovery. In the absence of further anthropogenic impacts, this is likely to continue over the next few years (Figure 1). In other coastal ecosystems, nutrient load reduction has been seen to lead to the recovery of seagrass meadows: in Tampa Bay (Florida) and Mondego Bay (Portugal), where seagrasses had almost disappeared, they now cover areas of 27 km^2^ and 1.6 km^2^, respectively [49].

Any comparison with other coastal transitional ecosystems must bear in mind that chemical values in the top layer of sediments may be influenced by several factors, including morphology, hydrodynamism, and seasonal fluctuations. However, in Óbidos Lagoon (Portugal), for example, a study showed that nutrient concentrations depended on distance from the Cal River mouth: TN ranged between 0.09 and 2.5 mg g^−1^ and TP ranged between 14 and 855 *μ*g g^−1^, being lowest on the seaward side [50]. Similarly, OC ranged from 0.16 to 29.0 mg g^−1^. In Corunna Lake (Australia) average TP and TN values ranged between 380 and 800 *μ*g g^−1^ and between 3.5 and 5.0 mg g^−1^, respectively [51]. The latter values are, on average, 3-4 times higher than the 2003 values for Venice (Figure 4) but are similar to the concentrations found in other Adriatic transitional systems such as the lagoons of Marano-Grado (TN: ca. 4.5 mg g^−1^, [52]) and the Po Delta (TN: ca. 1.48 mg g^−1^, [53]).

## 5. Conclusions

Venice Lagoon is an example of a transitional ecosystem in continuous evolution due to human intervention aimed not only at safeguarding but also at exploiting its resources. The reduction of macroalgal biomasses caused by climate change and other synergic factors [42], together with measures to reduce nutrient inputs [16], and the effect of sediment disturbance by anthropogenic activities such as clam harvesting and morphological intervention caused a significant decrease not only in OP but also in TN and OC in the top layer of sediments, with redistribution of fine sediments and disappearance of hot spot areas. As a result, the lagoon is still subject to significant anthropogenic pressures but the reduction in trophic status has allowed natural recolonization by seagrasses, so that many areas are moving towards a restoration of pristine conditions [18]. Lastly, the results of this paper represent a benchmark for the future, making it possible to highlight the effects of the ongoing regulation of water exchange at the lagoon inlets (including the use of mobile gates envisaged by the MoSE project), whose purpose is to prevent the frequent high water events affecting Venice and its lagoon.

## Figures and Tables

**Table 1:** Abiotic and biotic data measured in Venice Lagoon and sub-basins in 2003 survey (mean ± standard deviation (Std Dev), minimum (Min) and maximum (Max) values).

				Whole lagoon (W)	Northern basin (N)	Central basin (C)	Southern basin (S)
Depth	*D*	cm	Mean ± Std Dev	**117 ± 50.2**	**82.6 ± 25.7**	**136 ± 48.6**	**109 ± 49.7**
			Min ÷ Max	25.0 ÷ 270	45.0 ÷ 130	40.0 ÷ 270	25.0 ÷ 205

Temperature	*T*	°C	Mean ± Std Dev	**27.2 ± 1.93**	**28.3 ± 1.09**	**26.1 ± 1.84**	**28.0 ± 1.61**
			Min ÷ Max	22.7 ÷ 33.2	26.3 ÷ 30.2	22.7 ÷ 33.2	24.4 ÷ 30.9

pH	pH		Mean ± Std Dev	**8.20 ± 0.56**	**9.10 ± 0.14**	**7.73 ± 0.34**	**8.39 ± 0.35**
			Min ÷ Max	7.41 ÷ 9.35	8.85 ÷ 9.35	7.41 ÷ 9.20	7.83 ÷ 9.25

Salinity	*S*		Mean ± Std Dev	**33.5 ± 3.28**	**36.6 ± 4.28**	**31.2 ± 2.92**	**34.8 ± 1.58**
			Min ÷ Max	18.1 ÷ 43.1	24.2 ÷ 43.1	18.1 ÷ 35.6	27.8 ÷ 37.2

Secchi disk	SD	%	Mean ± Std Dev	**87.5 ± 21.0**	**86.7 ± 19.5**	**75.8 ± 24.7**	**98.1 ± 9.15**
			Min ÷ Max	20.0 ÷ 100	37.5 ÷ 100	20.0 ÷ 100	42.9 ÷ 100

Oxygen saturation	OS	%	Mean ± Std Dev	**116 ± 32.0**	**133 ± 36.6**	**116 ± 26.0**	**112 ± 34.5**
			Min ÷ Max	15.7 ÷ 239	95.5 ÷ 239	82.5 ÷ 224	15.7 ÷ 208

Sediment fine fraction	SFF	%	Mean ± Std Dev	**61.3 ± 28.0**	**88.7 ± 11.6**	**63.5 ± 27.6**	**52.6 ± 26.7**
			Min ÷ Max	1.91 ÷ 99.3	60.4 ÷ 99.3	1.91 ÷ 98.6	3.48 ÷ 98.0

Sediment dry density	SDD	g/cm^3^	Mean ± Std Dev	**0.95 ± 0.29**	**0.80 ± 0.19**	**1.04 ± 0.25**	**0.91 ± 0.32**
			Min ÷ Max	0.19 ÷ 1.47	0.49 ÷ 1.24	0.45 ÷ 1.47	0.19 ÷ 1.44

Macroalgal biomass	Mb	kg fwt/m^2^	Mean ± Std Dev	**0.41 ± 0.89**	**0.27 ± 0.55**	**0.18 ± 0.42**	**0.66 ± 1.17**
			Min ÷ Max	0.01 ÷ 7.50	0.01 ÷ 1.50	0.01 ÷ 3.00	0.01 ÷ 7.50

Seagrass biomass	Sb	kg fwt/m^2^	Mean ± Std Dev	**1.19 ± 2.16**	**0 ± 0**	**0.63 ± 1.57**	**1.99 ± 2.57**
			Min ÷ Max	0 ÷ 7.30	0 ÷ 0	0 ÷ 5.40	0 ÷ 7.30

			Observations	165	19	69	77

**Table 2:** Abiotic and biotic data measured in central part of Venice Lagoon in 1987, 1993, 1998, and 2003 surveys (mean ± standard deviation (Std Dev), minimum (Min) and maximum (Max) values).

			1987	1993	1998	2003	ANOVA
Temperature∗	°C	Mean ± Std Dev	**22.9 ± 2.14**	**25.7 ± 1.70**	**24.7 ± 2.72**	**25.7 ± 1.33**	<0.001
		Min ÷ Max	18.8 ÷ 27.8	22.9 ÷ 29.6	20.7 ÷ 29.7	22.7 ÷ 29.7	

pH∗		Mean ± Std Dev	**8.86 ± 0.38**	**8.48 ± 0.19**	**8.02 ± 0.13**	**7.65 ± 0.10**	<0.001
		Min ÷ Max	8.18 ÷ 9.54	8.21 ÷ 8.80	7.65 ÷ 8.27	7.41 ÷ 7.85	

Salinity		Mean ± Std Dev	**30.7 ± 1.72**	**31.4 ± 2.1**	**29.6 ± 3.16**	**30.9 ± 3.44**	0.08
		Min ÷ Max	25.5 ÷ 33.6	24.7 ÷ 34.8	22.2 ÷ 34.9	18.0 ÷ 35.4	

Oxygen saturation∗	%	Mean ± Std Dev	**282 ± 73.4**	**134 ± 29.0**	**116 ± 27.5**	**115 ± 22.3**	<0.001
		Min ÷ Max	159 ÷ 392	88.0 ÷ 214	70.0 ÷ 202	82.5 ÷ 181	

Sediment fine fraction	%	Mean ± Std Dev	**70.5 ± 27.1**		**60.4 ± 29.1**	**64.3 ± 29.8**	0.12
		Min ÷ Max	12.6 ÷ 98.7		11.1 ÷ 95.8	5.69 ÷ 98.2	

Macroalgal biomass∗	kg fwt/m^2^	Mean ± Std Dev	**5.86 ± 6.05**	**0.51 ± 1.50**	**0.20 ± 0.99**	**0.08 ± 0.18**	<0.001
		Min ÷ Max	0 ÷ 25.0	0 ÷ 7.50	0 ÷ 5.50	0 ÷ 0.80	

Total nitrogen∗	mg/cm^3^	Mean ± Std Dev	**1.19 ± 0.62**	**1.13 ± 0.50**	**0.86 ± 0.38**	**0.70 ± 0.27**	<0.001
		Min ÷ Max	0.22 ÷ 2.98	0.33 ÷ 2.62	0.13 ÷ 1.37	0.09 ÷ 1.15	

Inorganic phosphorus∗	*µ*g/cm^3^	Mean ± Std Dev	**282 ± 73.8**	**295 ± 73.8**	**320 ± 60.7**	**332 ± 83.2**	0.001
		Min ÷ Max	146 ÷ 474	141 ÷ 423	215 ÷ 466	216 ÷ 660	

Organic phosphorus∗	*µ*g/cm^3^	Mean ± Std Dev	**105 ± 43.3**	**65.0 ± 27.4**	**66.5 ± 27.0**	**53.2 ± 28.4**	<0.001
		Min ÷ Max	49.0 ÷ 246	27.0 ÷ 124	15.3 ÷ 117	3.02 ÷ 114	

Total phosphorus	*µ*g/cm^3^	Mean ± Std Dev	**387 ± 101**	**360 ± 82.6**	**386 ± 54.9**	**385 ± 79.7**	0.27
		Min ÷ Max	227 ÷ 720	184 ÷ 511	302 ÷ 534	295 ÷ 665	

Inorganic carbon	mg/cm^3^	Mean ± Std Dev	**66.4 ± 24.8**	**67.6 ± 27.0**	**68.0 ± 21.5**	**72.3 ± 22.7**	0.08
		Min ÷ Max	25.3 ÷ 117	22.0 ÷ 120	38.4 ÷ 121	28.1 ÷ 123	

Organic carbon∗	mg/cm^3^	Mean ± Std Dev	**8.85 ± 3.45**	**6.68 ± 3.30**	**10.7 ± 3.57**	**5.62 ± 2.64**	<0.001
		Min ÷ Max	2.72 ÷ 16.7	1.91 ÷ 14.2	4.30 ÷ 18.5	0.92 ÷ 12.7	

Total carbon	mg/cm^3^	Mean ± Std Dev	**75.2 ± 23.9**	**74.3 ± 25.9**	**78.7 ± 19.9**	**77.9 ± 22.0**	0.23
		Min ÷ Max	33.8 ÷ 124	25.6 ÷ 123	51.3 ÷ 127	33.6 ÷ 124	

		Observations	31	31	31	31	

∗Significant temporal fluctuations (ANOVA test, *p* < 0.05).
